# Crystal structures of tris­[1-oxo­pyridine-2-olato(1−)]silicon(IV) chloride chloro­form-*d*_1_ disolvate, tris­[1-oxo­pyridine-2-olato(1−)]silicon(IV) chloride aceto­nitrile unqu­anti­fied solvate, and *fac*-tris­[1-oxo­pyridine-2-thiol­ato(1−)]silicon(IV) chloride chloro­form-*d*_1_ disolvate

**DOI:** 10.1107/S2056989015022203

**Published:** 2015-11-28

**Authors:** Bradley M. Kraft, William W. Brennessel, Amy E. Ryan, Candace K. Benjamin

**Affiliations:** a Department of Chemistry, St. John Fisher College, Rochester, NY 14618, USA; b Department of Chemistry, University of Rochester, Rochester, NY 14627, USA

**Keywords:** crystal structure, silicon, pyridinone, pyridine *N*-oxide, pyri­thione

## Abstract

The cations in the title salts, [Si(OPO)_3_]Cl·2CDCl_3_, (I), [Si(OPO)_3_]Cl·*x*CH_3_CN, (II), and *fac*-[Si(OPTO)_3_]Cl·2CDCl_3_, (III) (OPO = 1-oxo-2-pyridin­one, C_5_H_4_NO_2_, and OPTO = 1-oxo-2-pyridine­thione, C_5_H_4_NOS), have distorted octa­hedral coordination spheres. The first two structures contain the same cation and anion, but different solvents of crystallization led to different solvates and packing arrangements. In structures (I) and (III), the silicon complex cations and chloride anions are well separated, while in (II), there are two C—H⋯Cl distances that fall just within the sum of the van der Waals radii of the C and Cl atoms. The pyridine portions of the OPO ligands in (I) and (II) are modeled as disordered with the planar flips of themselves [(I): 0.574 (15):0.426 (15), 0.696 (15):0.304 (15), and 0.621 (15):0.379 (15); (II): 0.555 (13):0.445 (13), 0.604 (14):0.396 (14) and 0.611 (13):0.389 (13)], demonstrating that both *fac* and *mer* isomers are co-crystallized. In (II), highly disordered solvent, located in two independent channels along [100], was unable to be modeled. Reflection contributions from this solvent were fixed and added to the calculated structure factors using the SQUEEZE [Spek (2015 ▸). *Acta Cryst.* C**71**, 9–18] function of program *PLATON*, which determined there to be 54 electrons in 225 Å^3^ accounted for per unit cell (25 electrons in 109 Å^3^ in one channel, and 29 electrons in 115 Å^3^ in the other). In (I) and (II), all species lie on general positions. In (III), all species are located along crystallographic threefold axes.

## Abstract 2

The homoleptic triply-ligated cation in two silyl chloride salt solvates of 1-oxo-2-pyridinone is found to co-crystallize as a mixture of *fac* and *mer* isomers. A related silyl cation of 1-oxo-2-pyridine­thione is found as the *fac* isomer.

## Chemical context   

Dissolution of silica by 1-hy­droxy-2-pyridinone (HOPO) at pH = 6 in aqueous solution has been shown to afford the cationic complex [Si(OPO)_3_]^+^, OPO = 1-oxo-2-pyridinone, which has been isolated as its chloride, tetra­chlorido­ferrate(III), and hexa­chlorido­stannate(IV) salts (Weiss & Harvey, 1964 ▸). Three other analogs, having tri­fluoro­methane­sulfonate, ethyl sulfate, and the isopropyl sulfate anion, were later synthesized by reaction of Si(OCH_3_)_4_ with HOPO with an appropriate acid and solvent and characterized by NMR spectroscopy (Tacke, Willeke & Penka, 2001 ▸). Our encounter with this stable cation occurred through an Si—C bond cleavage reaction involving (CH_2_)_3_Si(OPO)_2_ to yield (I) and through siloxane bond cleavage in Me_3_SiOSi(OPO)_2_Cl to form (II). We have additionally encountered the formation of the novel related sulfur analog, [Si(OPTO)_3_]^+^, OPTO = 1-oxo-2-pyridine­thione, also by an Si—C bond cleavage reaction involving (η^1^-all­yl)_2_Si(OPTO)Cl to afford (III). The driving force for the formation of the complexes is likely due to a combination of stabilizing lattice energy due to salt formation, ligand-binding strength enhanced by the chelate effect, and the added stabilization due to π-electron delocalization that occurs within the OPO and OPTO ligands upon chelation.
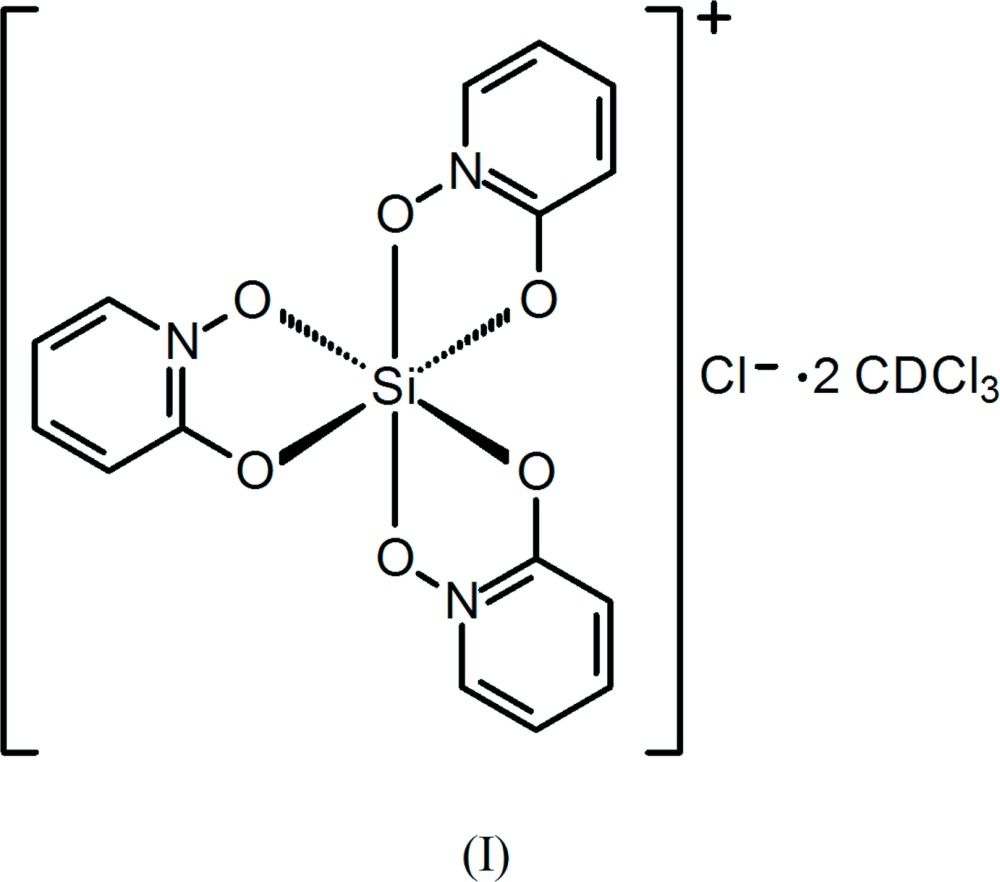
 
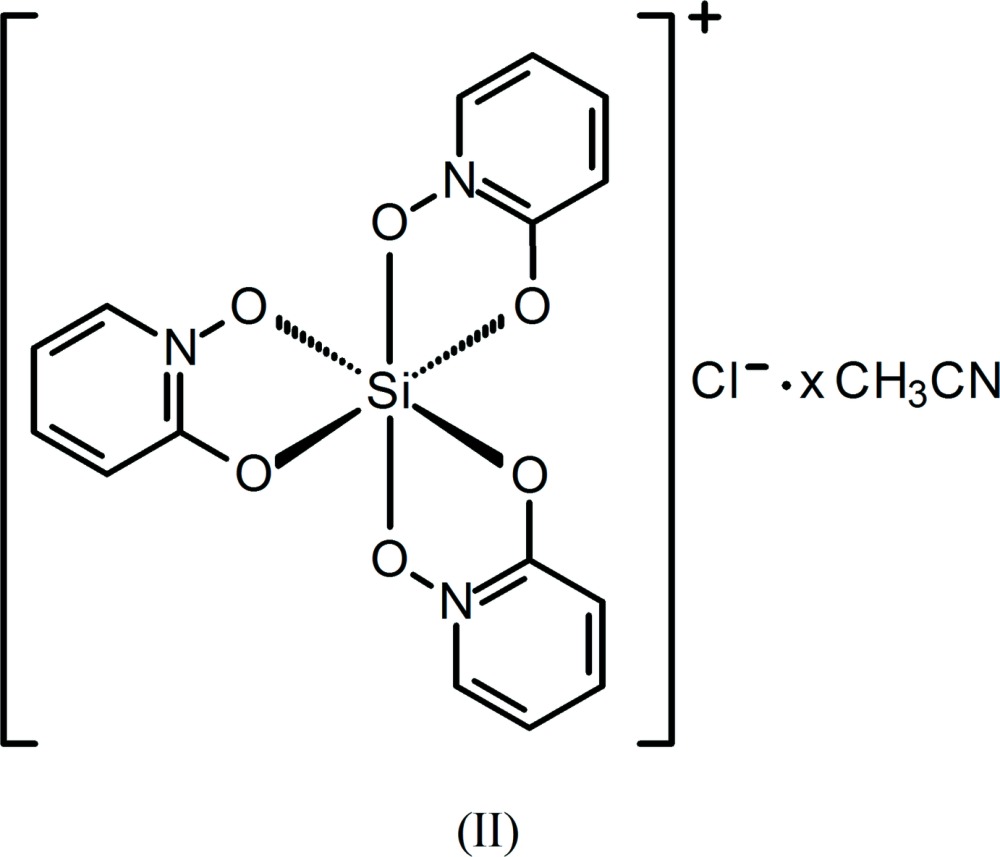
 
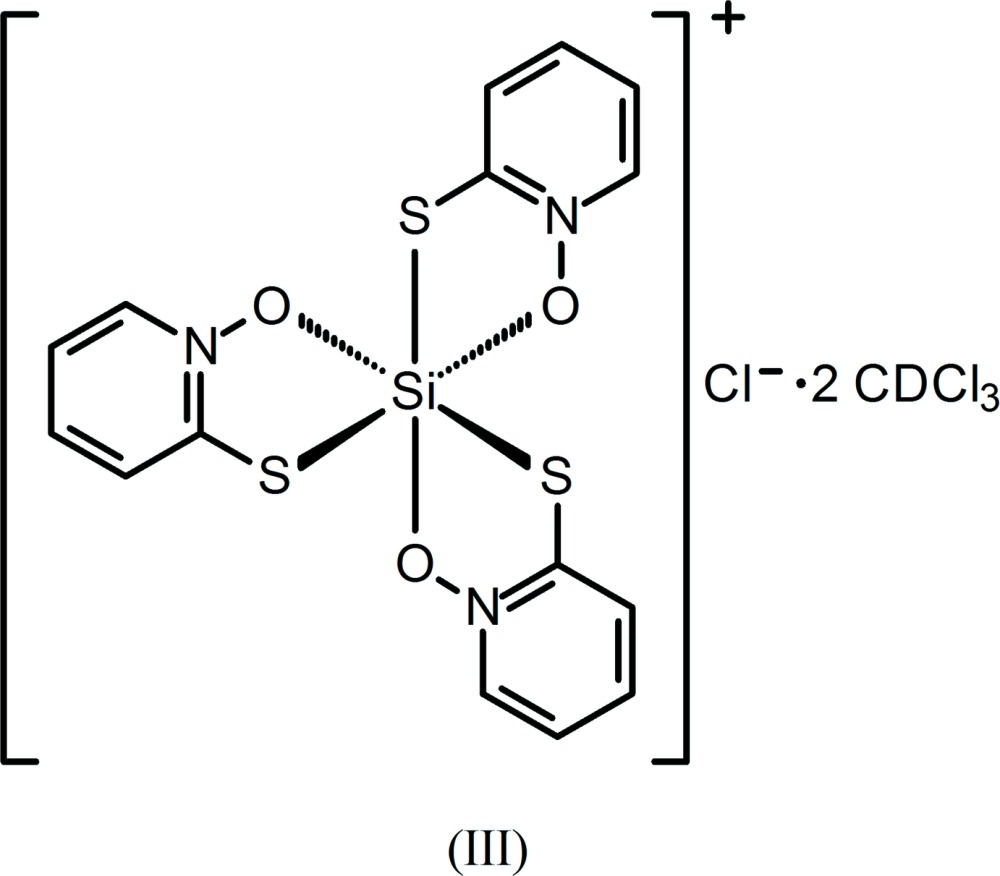


## Structural commentary   

**Figure 1:**
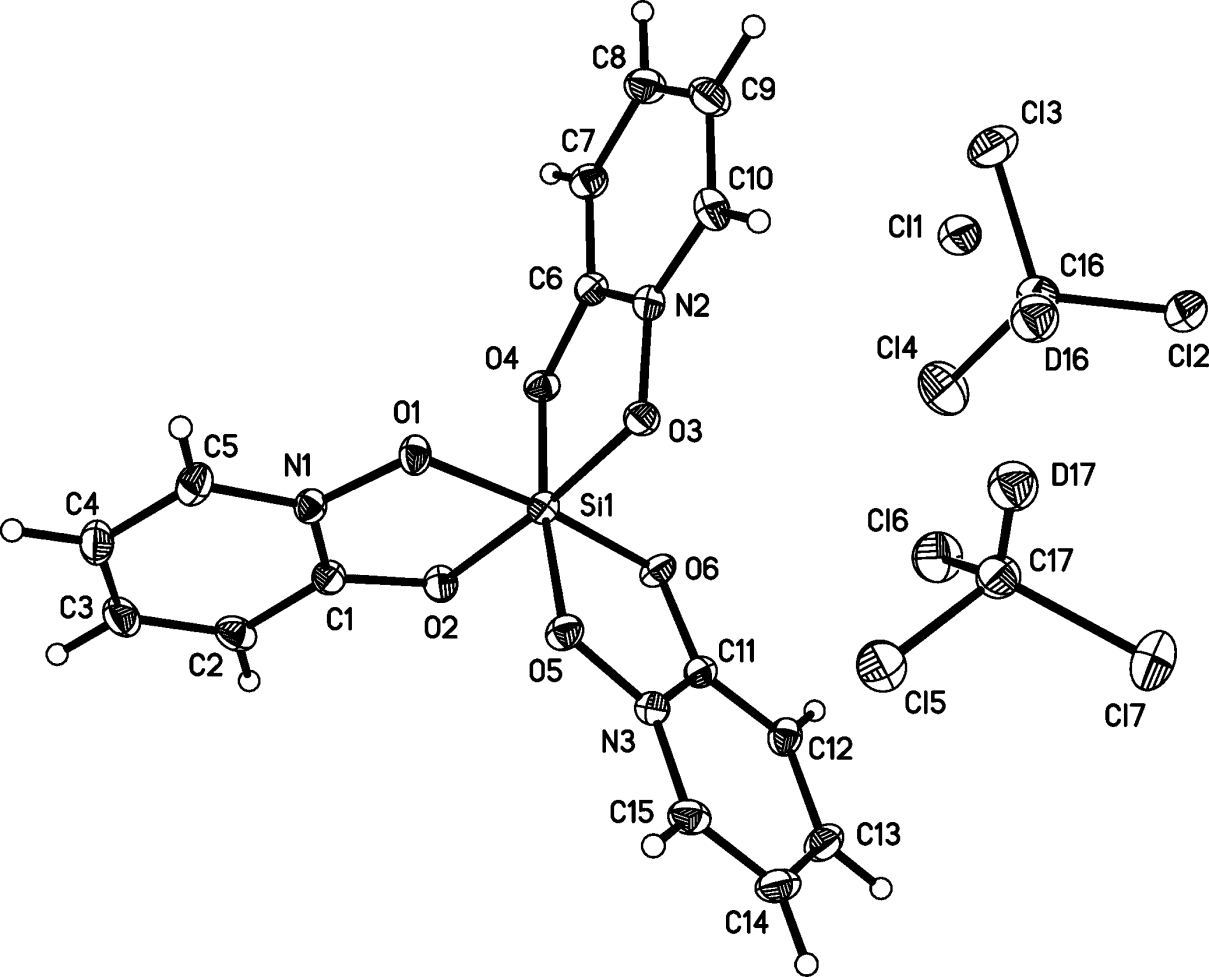
The structures of the molecular components in (I), with displacement ellipsoids drawn at the 50% probability level. The minor components of the ligand disorders are not shown.

The silicon atom in the structures of (I) and (II) is hexa­coordinate, chelated by three bidentate OPO ligands (Figs. 1 ▸ and 2 ▸). The isosteric ligands are disordered over the two possible coplanar orientations, such that each nitro­gen atom and its neighboring carbon atom are modeled as disordered with each other, which indicates both *fac* and *mer* isomers in each. The Si—O bond lengths in (I) and (II) span a narrow range from 1.7695 (10)–1.7774 (10) Å and 1.7727 (10)–1.7830 (10) Å, respectively (Tables 1 ▸ and 2 ▸). The O—Si—O ligand bite angles in (I) and (II) range from 86.99 (5)–87.24 (4)° and 87.28 (4)–87.38 (4)°, respectively. The *trans*-O—Si—O angles in (I) and (II) have a maximum deviation from ideal (*i.e.*, 180°) of 7.06 (5) and 5.98 (5)°, respectively. The planes formed by the O_2_Si chelate rings and the corresponding planar OPO ligand deviate from coplanarity by 9.98 (4), 4.96 (2), and 1.29 (2)° in (I) and by 4.91 (4), 2.15 (2), and 0.61 (4)° in (II).

**Figure 3:**
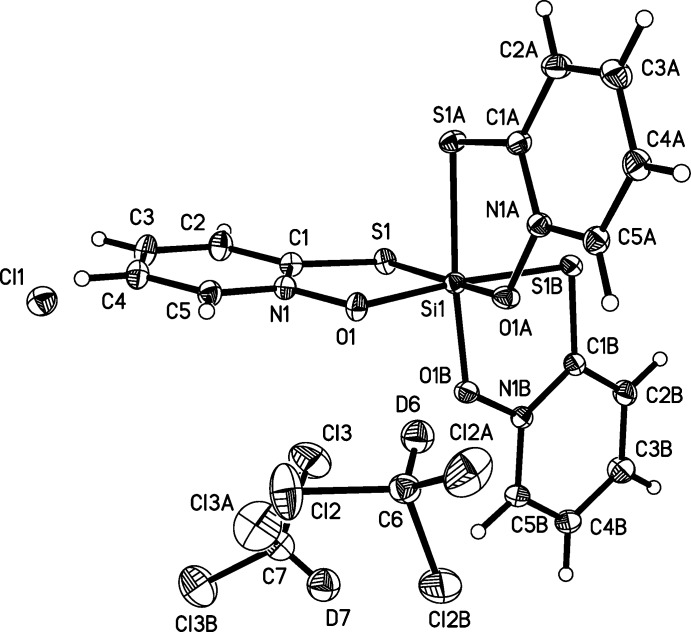
The structures of the molecular components and the Cl^−^ anion (III) with displacement ellipsoids drawn at the 50% probability level. All species lie along crystallographic threefold axes, and full mol­ecules are generated with the following symmetry codes. Si(OPTO)_3_^+^: (*y*, *z*, *x*) and (*z*, *x*, *y*); CDCl_3_ (containing atom C6): (*y*, *z*, *x*) and (*z*, *x*, *y*); CDCl_3_ (containing atom C7): (−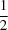 + *z*, 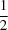 − *x*, 1 − *y*) and (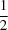 − *y*, 1 − *z*, 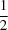 + *x*).

The cationic complex (III) is octa­hedral (Fig. 3 ▸) with the central Si atom being chelated by three OPTO ligands in a *facial* arrangement. The *trans*-O—Si—S angles deviate by 6.00 (5)° from ideal (only one unique value due to threefold symmetry, Table 3 ▸). The O—Si—S bite angles are 88.33 (4)°, ∼1° larger than those of the OPO structures. The Si—O distance is 1.7784 (14) Å, and compares similarly with those of (I) and (II) and is typical of Si—O single-bond lengths. The N—O bond is shorter than in the protonated HOPTO ligand [1.359 (2) *versus* 1.373 (2) Å; CSD refcode GIJCAD01, Bond & Jones, 1999 ▸, Cambridge Structural Database (CSD), Version 5.36, update No. 3, May 2015; Groom & Allen, 2014 ▸). Evidence of a π-electron delocalized structure is given by (1): the Si—S distance of 2.2654 (7) Å, which is similar to Si—S single-bond lengths in hexa­coordinate neutral thio­pheno­late complexes (range = 2.231–2.314 Å, CSD refcodes BOHQIZ, BOXQOV, BOXQUB, BOXRAI, WALTOU, WALTUA) and (2): the C—S bond length of 1.7184 (19) Å, which compares inter­mediately between the C=S double bond of HOPTO [1.693 (2) Å] and the mean C—S single bonds of 155 phenyl­thiols (C—S_avg_ 1.764 Å). However, all four C—C bond lengths in the pyridine ring are unchanged or slightly longer than those in HOPTO, which is inconsistent with the canonical pattern of bond shortening and lengthening that might be expected with π-electron delocalization. The OSSi chelate rings and the corresponding planar OPTO ligands are folded with a dihedral angle of 12.08 (3)°.

## Supra­molecular features   

In (II) there are two C—H⋯Cl distances that fall just within the sum of the van der Waals radii of the C and Cl atoms, 3.45 Å (Bondi, 1964 ▸). Atom C2 is 3.4206 (14) Å from atom Cl1 (symmetry operator: −*x*, −*y* + 1, −*z* + 2) and atom C10 is 3.4018 (18) Å from atom Cl1 (symmetry operator: *x*, *y* − 1, *z*).

## Database survey   

A CSD search (Groom & Allen, 2014 ▸) revealed one hit of the homoleptic cation in the form of [Si(OPO)_3_][CF_3_SO_3_]·0.5HOPO (CSD refcode QOXSIF; Tacke, Willeke & Penka, 2001 ▸). The Si—O bond lengths and bite angles in (I) and (II) are similar to those of QOXSIF. The dihedral angles formed between the O_2_Si chelate and OPO ligands are also similar to those of QOXSIF (9.39, 3.08, and 2.41°). Structures of monodentate organosilicon OPO complexes include Ph_3_Si(OPO)·Ph_3_Si(OH)·0.5*n*-pentane, Me_3_Si(OPO), and *t*Bu_2_Si(κ^1^-OPO)(κ^2^-OPO) (respective CSD refcodes NITRIT, NITROZ, and NITSOA; Kraft & Brennessel, 2014 ▸), and of bidentate organosilicon OPO complexes include Ph_2_Si(OPO)_2_, Me_2_Si(OPO)Cl, Ph_3_Si(OPO), Me_2_Si(OPO)_2_, Et_2_Si(OPO)_2_, *i*Pr_2_Si(OPO)_2_, *t*Bu_2_Si(κ^1^-OPO)(κ^2^-OPO), and (CH_2_)_3_Si(OPO)_2_ (respective CSD refcodes NISMIN, NISMOT, NITRUF, NITSAM, NITSEQ, NITSOA, NITSIU, and NITSUG; Kraft & Brennessel, 2014 ▸), and [Si(OPO)_2_(μ-CH_2_CH_2_SCH_2_C(=O)O)]_2_·2CH_3_CN and [O(CH_2_)_3_]Si(OPO)_2_ (respective CSD refcodes UBUWET and UBUWIX; Tacke, Burschka *et al.*, 2001 ▸). (I) and (II) have 0.06–0.17 Å shorter Si—O bond lengths and 3–5° larger ligand bite angles than those in chelated *R*_2_Si(OPO)_2_ (*R* = alkyl, phen­yl) complexes, indicating a stronger chelate presumably due, in part, to their cationic character. As a result of C/N site disorders, the N—O, C—O, and C—N bond lengths are unreliable in providing evidence of π-electron delocalization. Only small changes (± ∼0.02–0.06 Å) or no change (in C3—C4) in the distances of alternating long and short C—C bonds in the pyridine ring are observed compared with the more localized π-bonding structure of the free HOPO ligand (CSD refcode JEMJUG; Ballesteros *et al.*, 1990 ▸). The Si—O bond lengths in (I) and (II) are similar to those of other cationic SiO_6_ cores (CSD refcodes CUZKOX: Ueyama *et al.*, 1985 ▸; EJOBUB: Sarkar *et al.*, 2011 ▸, JAZPIK: Pal *et al.*, 2005 ▸; PUMBUU: Kira *et al.*, 1998 ▸; VILLUX: Thewalt & Link, 1991 ▸). There are two other non-silicon homoleptic *M*(OPO)_3_ (*M* = Fe, Co) structures known (CSD refcodes DAGZOA and DAGZIU01; Scarrow *et al.*, 1985 ▸).

There are currently no structurally characterized silicon OPTO complexes. Other triply ligated homoleptic *M*(OPTO)_3_ structures are: *M* = Cr (CSD refcode ZUZWEW; Wen *et al.*, 1996 ▸), *M* = Mn (IFOPAU: Liaw *et al.*, 2002 ▸; SUJYEB: Manivannan *et al.*, 1993 ▸), *M* = Fe (PEDEKO; Hu *et al.*, 1993 ▸), *M* = Co (VOGHAA: Hu *et al.*, 1991 ▸; SUJYAX, SUJYEB: Manivannan *et al.*, 1993 ▸; WINFUU, WINGAB: Xu *et al.*, 1995 ▸; ROLQUE: Tong *et al.*, 2001 ▸; UGUCUU: Fang *et al.*, 2002 ▸), *M* = In, Tl (JIVQAG, JIVQE; Rodríguez *et al.*, 1998 ▸), *M* = Bi (BEHDOI; Niu *et al.*, 2003 ▸).

There are currently nine CSD entries for other group 14 complexes containing an OPTO ligand, all with tin: CSD refcodes ENEWEZ, ENEWID, FOFNAP/FOFNAP10, FOTBOF, IMECAE, IMECEI, IMECIM, and YEDVEI.

## Synthesis and crystallization   

**[Si(OPO)_3_]Cl·2CDCl_3_ (I)**: (CH_2_)_3_Si(OPO)_2_ was prepared according to the literature method (Kraft & Brennessel, 2014 ▸). (CH_2_)_3_Si(OPO)_2_ was heated in an oil bath at 463 K for 3 days in CDCl_3_ upon which crystals of (I) deposited.

**[Si(OPO)_3_]Cl·** ***x*** **CH_3_CN (II)**: A solution of Me_3_Si(OPO) (0.183 g, 1.00 mmol) in 8 ml of CH_3_CN was added to a solution of Me_3_SiOSiCl_3_ (98 µ*L*, d = 1.14 g/ml, 0.50 mmol) in 4 ml of CH_3_CN. Me_3_SiOSi(OPO)_2_Cl is formed as an intermediate. Allowing the solution to stand undisturbed for one day resulted in precipitation of colorless crystals of (II) (0.090 g) which were isolated by filtration. Evidence for the presence of *fac* and *mer* isomers was given by the presence of closely spaced OPO resonances in the ^13^C NMR spectrum in accord with those reported in the literature (Tacke, Willeke & Penka, 2001 ▸). The synthesis, isolation, and characterization of Me_3_SiOSi(OPO)_2_Cl will be reported elsewhere.

**[Si(OPTO)_3_]Cl·2CDCl_3_ (III)**: Crystals of (III) deposited from a solution of (η^1^-all­yl)_2_Si(OPTO)Cl in CDCl_3_ upon standing for one year at room temperature in the dark. The synthesis of (η^1^-all­yl)_2_Si(OPTO)Cl will be published elsewhere.

## Refinement   

Crystal data, data collection and structure refinement details are summarized in Table 4 ▸. The pyridine portions of the OPO ligands in (I) and (II) are modeled as disordered with the coplanar flips of themselves [0.574 (15):0.426 (15), 0.696 (15):0.304 (15), and 0.621 (15):0.379 (15) for rings containing N1, N2, and N3, respectively, in (I), and 0.555 (13):0.445 (13), 0.604 (14):0.396 (14) and 0.611 (13):0.389 (13) for rings containing N1, N2, and N3 in (II)]. The disorders were modeled by refining the nitro­gen/carbon ratios in each of the specific sites while using a common variable for pairs of sites on the same ligand. Atoms at each of these sites were constrained to be isopositional and to have equivalent anisotropic displacement parameters.

In (II) highly disordered solvent, located in two independent channels along [100], was unable to be modeled. Reflection contributions from this solvent were fixed and added to the calculated structure factors using the SQUEEZE (Spek, 2015 ▸) function of the *PLATON* program, which determined there to be 54 electrons in 225 Å^3^ accounted for per unit cell (25 electrons in 109 Å^3^ in one channel, and 29 electrons in 115 Å^3^ in the other). Although the exact amount of solvent was unknown, the only solvent involved in the reaction was aceto­nitrile and both starting materials were confirmed by ^1^H NMR to be unsolvated. Thus the structure is represented as an aceto­nitrile solvate of unknown amount. Because no solvent was included in the atom list or mol­ecular formula for (II), all calculated qu­anti­ties that derive from the mol­ecular formula [*e.g.*, F(000), density, mol­ecular weight, *etc*.] are known to be incorrect.

D and H atoms were placed geometrically and treated as riding atoms: methine, C—D = 1.00 Å, and aromatic, C—H = 0.95 Å, with *U*_iso_(H/D) = 1.2*U*_eq_(C).

## Supplementary Material

Crystal structure: contains datablock(s) I, II, III, global. DOI: 10.1107/S2056989015022203/hg5464sup1.cif

Structure factors: contains datablock(s) I. DOI: 10.1107/S2056989015022203/hg5464Isup2.hkl

Structure factors: contains datablock(s) II. DOI: 10.1107/S2056989015022203/hg5464IIsup3.hkl

Structure factors: contains datablock(s) III. DOI: 10.1107/S2056989015022203/hg5464IIIsup4.hkl

CCDC references: 1438045, 1438046, 1438047

Additional supporting information:  crystallographic information; 3D view; checkCIF report

## Figures and Tables

**Figure 2:**
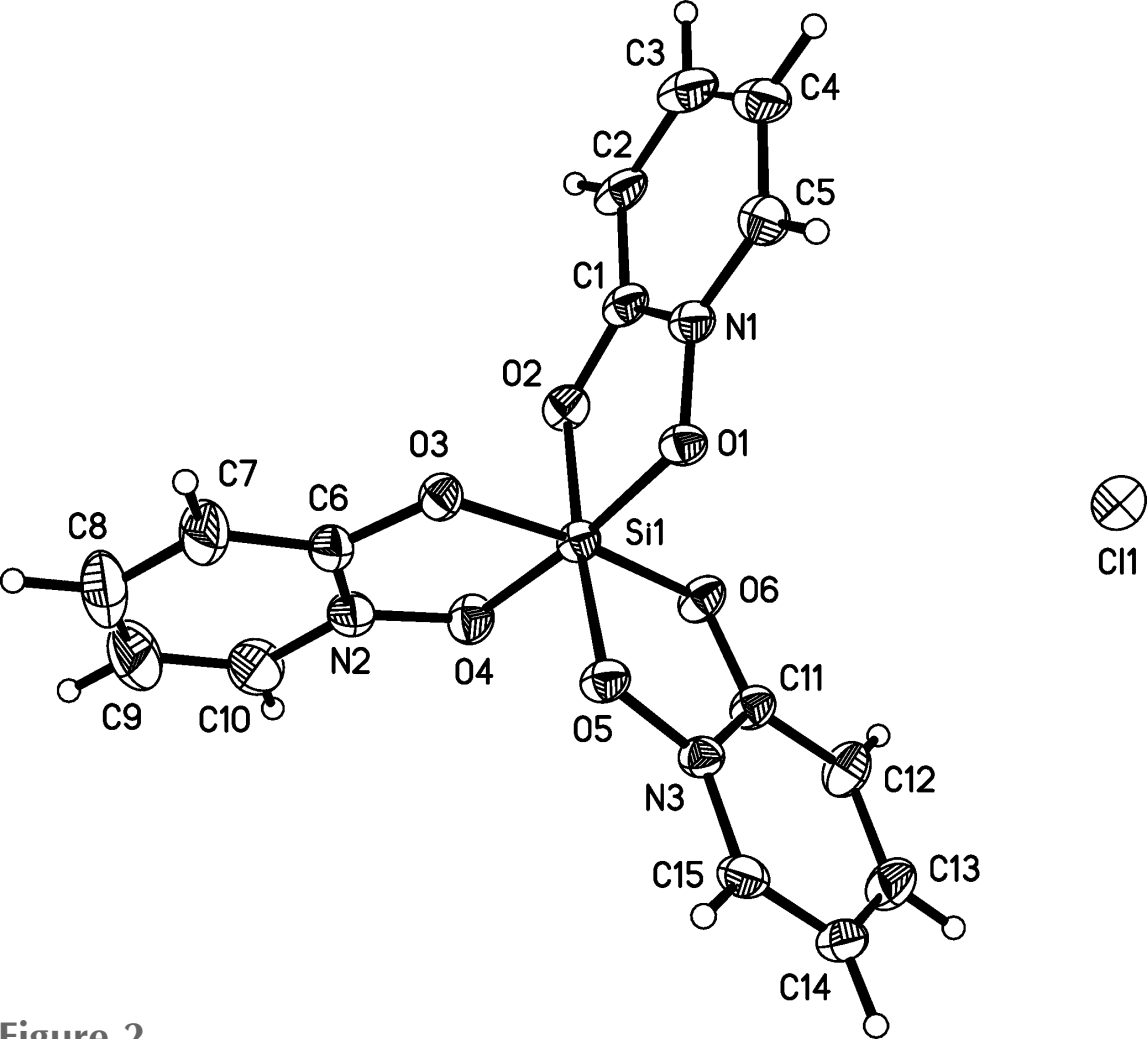
The molecular structure of the cation and the Cl^−^ anion in (II), with displacement ellipsoids drawn at the 50% probability level. The minor components of the ligand disorders and the unmodeled solvent (see text) are not shown.

**Table 1:** Selected bond lengths (Å) for (I)

Si1—O3	1.7695 (10)	Si1—O1	1.7767 (10)
Si1—O2	1.7727 (10)	Si1—O4	1.7773 (10)
Si1—O6	1.7736 (10)	Si1—O5	1.7774 (10)

**Table 2:** Selected bond lengths (Å) for (II)

Si1—O1	1.7727 (10)	Si1—O5	1.7803 (10)
Si1—O6	1.7729 (9)	Si1—O4	1.7808 (10)
Si1—O3	1.7782 (9)	Si1—O2	1.7830 (10)

**Table 3:** Selected geometric parameters (Å, °) for (III)

Si1—O1	1.7784 (14)	Si1—S1	2.2654 (7)
			
O1—Si1—S1	88.33 (4)	O1—Si1—S1^i^	174.00 (5)

Symmetry code: (i) 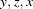.

**Table 4:** Experimental details

	(I)	(II)	(III)
Crystal data			
Chemical formula	C_15_H_12_N_3_O_6_Si^+^·Cl^−^·2CDCl_3_	C_15_H_12_N_3_O_6_Si^+^·Cl^−^	C_15_H_12_N_3_O_3_S_3_Si^+^·Cl^−^·2CDCl_3_
*M*_r_	634.56	393.82	682.74
Crystal system, space group	Monoclinic, *P*2_1_/*n*	Triclinic, *P* 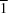	Cubic, *P*2_1_3
Temperature (K)	100	100	100
*a*, *b*, *c* (Å)	13.5133 (7), 13.5039 (7), 13.7752 (7)	6.8347 (7), 11.1232 (12), 13.1513 (14)	13.9483 (12), 13.9483 (12), 13.9483 (12)
α, β, γ (°)	90, 101.866 (1), 90	90.479 (2), 93.269 (2), 102.356 (2)	90, 90, 90
*V* (Å^3^)	2460.0 (2)	974.85 (18)	2713.7 (7)
*Z*	4	2	4
Radiation type	Mo *K*α	Mo *K*α	Mo *K*α
μ (mm^−1^)	0.90	0.29	1.03
Crystal size (mm)	0.20 × 0.18 × 0.16	0.30 × 0.30 × 0.24	0.18 × 0.18 × 0.18

Data collection			
Diffractometer	Bruker SMART APEXII CCD Platform	Bruker SMART APEXII CCD Platform	Bruker SMART APEXII CCD Platform
Absorption correction	Multi-scan (*SADABS*; Sheldrick, 2014 ▸)	Multi-scan (*SADABS*; Sheldrick, 2014 ▸)	Multi-scan (*SADABS*; Sheldrick, 2014 ▸)
*T*_min_, *T*_max_	0.667, 0.748	0.645, 0.748	0.681, 0.748
No. of measured, independent and observed [*I* > 2σ(*I*)] reflections	61259, 13615, 9035	27002, 10311, 6677	66318, 5067, 4360
*R*_int_	0.051	0.037	0.059
(sin θ/λ)_max_ (Å^−1^)	0.880	0.875	0.877

Refinement			
*R*[*F* ^2^ > 2σ(*F* ^2^)], *wR*(*F* ^2^), *S*	0.044, 0.117, 1.04	0.050, 0.132, 1.03	0.037, 0.088, 1.03
No. of reflections	13615	10311	5067
No. of parameters	310	238	103
H-atom treatment	H-atom parameters constrained	H-atom parameters constrained	H-atom parameters constrained
Δρ_max_, Δρ_min_ (e Å^−3^)	0.81, −0.85	0.45, −0.45	0.88, −0.67
Absolute structure	–	–	Flack *x* determined using 1775 quotients [(*I* ^+^)−(*I* ^−^)]/[(*I* ^+^)+(*I* ^−^)] (Parsons *et al.*, 2013 ▸)
Absolute structure parameter	–	–	−0.018 (18)

Computer programs: *APEX2* (Bruker, 2014 ▸), *SAINT* (Bruker, 2013 ▸), *SIR2011* (Burla *et al.*, 2012 ▸), *SHELXL2012* (Sheldrick, 2015 ▸), *SHELXL2014* (Sheldrick, 2015 ▸) and *SHELXTL* (Sheldrick, 2008 ▸).

## supplementary crystallographic information

### (I) Tris[1-oxopyridine-2-olato(1-)]silicon(IV) chloride chloroform-d1 disolvate Crystal data

**Table:**

C_15_H_12_N_3_O_6_Si^+^·Cl^−^·2CDCl_3_	*F*(000) = 1272
*M**_r_* = 634.56	*D*_x_ = 1.713 Mg m^−^^3^
Monoclinic, *P*2_1_/*n*	Mo *K*α radiation, λ = 0.71073 Å
*a* = 13.5133 (7) Å	Cell parameters from 3986 reflections
*b* = 13.5039 (7) Å	θ = 2.4–37.0°
*c* = 13.7752 (7) Å	µ = 0.90 mm^−^^1^
β = 101.866 (1)°	*T* = 100 K
*V* = 2460.0 (2) Å^3^	Block, pale red-yellow
*Z* = 4	0.20 × 0.18 × 0.16 mm

### (I) Tris[1-oxopyridine-2-olato(1-)]silicon(IV) chloride chloroform-d1 disolvate Data collection

**Table:**

Bruker SMART APEXII CCD Platform diffractometer	13615 independent reflections
Radiation source: sealed tube	9035 reflections with *I* > 2σ(*I*)
Graphite monochromator	*R*_int_ = 0.051
area detector, ω scans per φ	θ_max_ = 38.7°, θ_min_ = 1.9°
Absorption correction: multi-scan (SADABS; Sheldrick, 2014)	*h* = −23→23
*T*_min_ = 0.667, *T*_max_ = 0.748	*k* = −23→23
61259 measured reflections	*l* = −24→23

### (I) Tris[1-oxopyridine-2-olato(1-)]silicon(IV) chloride chloroform-d1 disolvate Refinement

**Table:**

Refinement on *F*^2^	Primary atom site location: structure-invariant direct methods
Least-squares matrix: full	Secondary atom site location: difference Fourier map
*R*[*F*^2^ > 2σ(*F*^2^)] = 0.044	Hydrogen site location: inferred from neighbouring sites
*wR*(*F*^2^) = 0.117	H-atom parameters constrained
*S* = 1.04	*w* = 1/[σ^2^(*F*_o_^2^) + (0.0513*P*)^2^ + 0.4945*P*] where *P* = (*F*_o_^2^ + 2*F*_c_^2^)/3
13615 reflections	(Δ/σ)_max_ = 0.001
310 parameters	Δρ_max_ = 0.81 e Å^−^^3^
0 restraints	Δρ_min_ = −0.85 e Å^−^^3^

### (I) Tris[1-oxopyridine-2-olato(1-)]silicon(IV) chloride chloroform-d1 disolvate Special details

**Table:**

Geometry. All e.s.d.'s (except the e.s.d. in the dihedral angle between two l.s. planes) are estimated using the full covariance matrix. The cell e.s.d.'s are taken into account individually in the estimation of e.s.d.'s in distances, angles and torsion angles; correlations between e.s.d.'s in cell parameters are only used when they are defined by crystal symmetry. An approximate (isotropic) treatment of cell e.s.d.'s is used for estimating e.s.d.'s involving l.s. planes.

### (I) Tris[1-oxopyridine-2-olato(1-)]silicon(IV) chloride chloroform-d1 disolvate Fractional atomic coordinates and isotropic or equivalent isotropic displacement parameters (Å^2^)

**Table:**

	*x*	*y*	*z*	*U*_iso_*/*U*_eq_	Occ. (<1)
Si1	0.23776 (3)	0.49175 (3)	0.47775 (3)	0.01307 (7)	
O1	0.10428 (7)	0.47729 (7)	0.44717 (7)	0.01561 (17)	
O2	0.24538 (7)	0.36073 (7)	0.48242 (7)	0.01631 (17)	
O3	0.22003 (7)	0.62136 (7)	0.46597 (7)	0.01524 (16)	
O4	0.25223 (7)	0.49211 (7)	0.35240 (7)	0.01496 (16)	
O5	0.23135 (7)	0.50555 (7)	0.60463 (7)	0.01575 (17)	
O6	0.37121 (7)	0.49674 (7)	0.51655 (7)	0.01554 (17)	
N1	0.07670 (9)	0.38170 (8)	0.45015 (8)	0.0137 (2)	0.574 (15)
N2	0.21142 (9)	0.65110 (8)	0.37143 (8)	0.0146 (2)	0.696 (15)
N3	0.32336 (9)	0.51079 (9)	0.66292 (8)	0.0149 (2)	0.621 (15)
N1'	0.15514 (9)	0.31791 (9)	0.47064 (9)	0.0150 (2)	0.426 (15)
N2'	0.22799 (9)	0.57936 (9)	0.30881 (9)	0.0146 (2)	0.304 (15)
N3'	0.40015 (9)	0.50504 (9)	0.61468 (8)	0.0135 (2)	0.379 (15)
C1	0.15514 (9)	0.31791 (9)	0.47064 (9)	0.0150 (2)	0.574 (15)
C1'	0.07670 (9)	0.38170 (8)	0.45015 (8)	0.0137 (2)	0.426 (15)
C2	0.13816 (12)	0.21819 (10)	0.47797 (10)	0.0194 (2)	
H2	0.1929	0.1728	0.4936	0.023*	
C3	0.03977 (12)	0.18600 (11)	0.46201 (11)	0.0228 (3)	
H3	0.0263	0.1173	0.4663	0.027*	
C4	−0.04059 (12)	0.25241 (12)	0.43964 (11)	0.0228 (3)	
H4	−0.1083	0.2291	0.4289	0.027*	
C5	−0.02127 (10)	0.35157 (11)	0.43323 (10)	0.0189 (2)	
H5	−0.0750	0.3980	0.4174	0.023*	
C6	0.22799 (9)	0.57936 (9)	0.30881 (9)	0.0146 (2)	0.696 (15)
C6'	0.21142 (9)	0.65110 (8)	0.37143 (8)	0.0146 (2)	0.304 (15)
C7	0.21831 (10)	0.59879 (10)	0.20932 (10)	0.0177 (2)	
H7	0.2293	0.5485	0.1645	0.021*	
C8	0.19215 (11)	0.69353 (11)	0.17687 (11)	0.0208 (3)	
H8	0.1833	0.7087	0.1083	0.025*	
C9	0.17853 (11)	0.76731 (11)	0.24355 (11)	0.0215 (3)	
H9	0.1625	0.8328	0.2205	0.026*	
C10	0.18812 (11)	0.74589 (10)	0.34229 (11)	0.0187 (2)	
H10	0.1788	0.7954	0.3886	0.022*	
C11	0.40015 (9)	0.50504 (9)	0.61468 (8)	0.0135 (2)	0.621 (15)
C11'	0.32336 (9)	0.51079 (9)	0.66292 (8)	0.0149 (2)	0.379 (15)
C12	0.49892 (10)	0.50876 (10)	0.66439 (10)	0.0169 (2)	
H12	0.5528	0.5049	0.6298	0.020*	
C13	0.51771 (11)	0.51829 (11)	0.76596 (10)	0.0211 (3)	
H13	0.5854	0.5204	0.8025	0.025*	
C14	0.43758 (12)	0.52481 (11)	0.81544 (10)	0.0214 (3)	
H14	0.4510	0.5316	0.8856	0.026*	
C15	0.33966 (11)	0.52145 (10)	0.76344 (10)	0.0182 (2)	
H15	0.2847	0.5264	0.7964	0.022*	
Cl1	0.20123 (3)	0.95652 (2)	0.49660 (2)	0.01944 (6)	
C16	0.47965 (12)	0.85178 (11)	0.41139 (11)	0.0230 (3)	
D16	0.4348	0.8696	0.4581	0.028*	
Cl2	0.59065 (3)	0.92375 (3)	0.44147 (3)	0.02772 (8)	
Cl3	0.41495 (3)	0.87758 (4)	0.29021 (3)	0.03465 (10)	
Cl4	0.50920 (4)	0.72498 (3)	0.42591 (4)	0.03726 (10)	
C17	0.35128 (11)	0.83844 (11)	0.70226 (11)	0.0211 (3)	
D17	0.3135	0.8897	0.6569	0.025*	
Cl5	0.26553 (3)	0.77821 (3)	0.76321 (3)	0.02750 (8)	
Cl6	0.40505 (3)	0.75370 (3)	0.63070 (3)	0.02850 (8)	
Cl7	0.44653 (3)	0.89800 (3)	0.78891 (3)	0.02811 (8)	

### (I) Tris[1-oxopyridine-2-olato(1-)]silicon(IV) chloride chloroform-d1 disolvate Atomic displacement parameters (Å^2^)

**Table:**

	*U*^11^	*U*^22^	*U*^33^	*U*^12^	*U*^13^	*U*^23^
Si1	0.01221 (14)	0.01345 (15)	0.01315 (14)	0.00003 (11)	0.00173 (11)	−0.00084 (11)
O1	0.0135 (4)	0.0115 (4)	0.0211 (4)	−0.0006 (3)	0.0019 (3)	−0.0015 (3)
O2	0.0136 (4)	0.0141 (4)	0.0207 (4)	0.0013 (3)	0.0024 (3)	0.0007 (3)
O3	0.0192 (4)	0.0140 (4)	0.0124 (4)	−0.0008 (3)	0.0031 (3)	−0.0008 (3)
O4	0.0167 (4)	0.0136 (4)	0.0138 (4)	0.0025 (3)	0.0014 (3)	−0.0003 (3)
O5	0.0124 (4)	0.0209 (4)	0.0140 (4)	0.0001 (3)	0.0027 (3)	0.0001 (3)
O6	0.0127 (4)	0.0227 (5)	0.0109 (4)	−0.0005 (3)	0.0015 (3)	−0.0016 (3)
N1	0.0142 (5)	0.0124 (4)	0.0142 (5)	−0.0003 (4)	0.0023 (4)	−0.0016 (4)
N2	0.0149 (5)	0.0141 (5)	0.0145 (5)	−0.0018 (4)	0.0020 (4)	−0.0007 (4)
N3	0.0163 (5)	0.0142 (5)	0.0137 (5)	−0.0006 (4)	0.0023 (4)	0.0006 (4)
N1'	0.0150 (5)	0.0138 (5)	0.0158 (5)	0.0003 (4)	0.0025 (4)	−0.0002 (4)
N2'	0.0142 (5)	0.0151 (5)	0.0140 (5)	0.0000 (4)	0.0018 (4)	−0.0004 (4)
N3'	0.0141 (5)	0.0136 (5)	0.0124 (5)	0.0004 (4)	0.0017 (4)	−0.0002 (4)
C1	0.0150 (5)	0.0138 (5)	0.0158 (5)	0.0003 (4)	0.0025 (4)	−0.0002 (4)
C1'	0.0142 (5)	0.0124 (4)	0.0142 (5)	−0.0003 (4)	0.0023 (4)	−0.0016 (4)
C2	0.0271 (7)	0.0134 (5)	0.0180 (6)	0.0015 (5)	0.0054 (5)	0.0013 (4)
C3	0.0331 (8)	0.0163 (6)	0.0188 (6)	−0.0086 (5)	0.0053 (5)	−0.0004 (5)
C4	0.0226 (6)	0.0255 (7)	0.0199 (6)	−0.0105 (5)	0.0037 (5)	−0.0025 (5)
C5	0.0138 (5)	0.0240 (6)	0.0188 (6)	−0.0017 (5)	0.0031 (4)	−0.0029 (5)
C6	0.0142 (5)	0.0151 (5)	0.0140 (5)	0.0000 (4)	0.0018 (4)	−0.0004 (4)
C6'	0.0149 (5)	0.0141 (5)	0.0145 (5)	−0.0018 (4)	0.0020 (4)	−0.0007 (4)
C7	0.0176 (5)	0.0206 (6)	0.0148 (5)	0.0006 (5)	0.0033 (4)	−0.0002 (4)
C8	0.0211 (6)	0.0234 (6)	0.0177 (6)	−0.0003 (5)	0.0037 (5)	0.0048 (5)
C9	0.0236 (6)	0.0161 (6)	0.0249 (7)	0.0003 (5)	0.0050 (5)	0.0055 (5)
C10	0.0199 (6)	0.0137 (5)	0.0229 (6)	−0.0014 (4)	0.0057 (5)	−0.0001 (4)
C11	0.0141 (5)	0.0136 (5)	0.0124 (5)	0.0004 (4)	0.0017 (4)	−0.0002 (4)
C11'	0.0163 (5)	0.0142 (5)	0.0137 (5)	−0.0006 (4)	0.0023 (4)	0.0006 (4)
C12	0.0138 (5)	0.0169 (5)	0.0195 (6)	0.0002 (4)	0.0019 (4)	−0.0009 (4)
C13	0.0207 (6)	0.0205 (6)	0.0186 (6)	0.0009 (5)	−0.0043 (5)	−0.0007 (5)
C14	0.0305 (7)	0.0193 (6)	0.0127 (5)	0.0022 (5)	0.0003 (5)	0.0003 (4)
C15	0.0245 (6)	0.0172 (5)	0.0136 (5)	0.0009 (5)	0.0054 (5)	0.0008 (4)
Cl1	0.02314 (15)	0.01732 (13)	0.01640 (13)	−0.00044 (11)	0.00069 (11)	0.00007 (10)
C16	0.0255 (7)	0.0195 (6)	0.0223 (6)	0.0015 (5)	0.0013 (5)	0.0020 (5)
Cl2	0.02631 (17)	0.02598 (17)	0.02676 (17)	−0.00130 (13)	−0.00412 (14)	0.00140 (13)
Cl3	0.02946 (19)	0.0446 (2)	0.02482 (18)	−0.00543 (17)	−0.00619 (15)	0.00552 (16)
Cl4	0.0459 (3)	0.01883 (17)	0.0466 (3)	0.00556 (16)	0.0084 (2)	−0.00106 (16)
C17	0.0231 (6)	0.0175 (6)	0.0213 (6)	0.0024 (5)	0.0015 (5)	−0.0001 (5)
Cl5	0.02676 (17)	0.02573 (17)	0.02922 (18)	−0.00212 (14)	0.00391 (14)	0.00239 (14)
Cl6	0.0362 (2)	0.02194 (16)	0.02728 (18)	0.00365 (14)	0.00644 (15)	−0.00488 (13)
Cl7	0.02268 (16)	0.02851 (18)	0.03154 (19)	−0.00142 (13)	0.00187 (14)	−0.01005 (14)

### (I) Tris[1-oxopyridine-2-olato(1-)]silicon(IV) chloride chloroform-d1 disolvate Geometric parameters (Å, º)

**Table:**

Si1—O3	1.7695 (10)	C5—H5	0.9500
Si1—O2	1.7727 (10)	C7—C8	1.377 (2)
Si1—O6	1.7736 (10)	C7—H7	0.9500
Si1—O1	1.7767 (10)	C8—C9	1.393 (2)
Si1—O4	1.7773 (10)	C8—H8	0.9500
Si1—O5	1.7774 (10)	C9—C10	1.370 (2)
O1—N1	1.3465 (15)	C9—H9	0.9500
O2—N1'	1.3290 (15)	C10—H10	0.9500
O3—N2	1.3448 (14)	C12—C13	1.376 (2)
O4—N2'	1.3323 (15)	C12—H12	0.9500
O5—N3	1.3361 (15)	C13—C14	1.396 (2)
O6—N3'	1.3325 (15)	C13—H13	0.9500
N1—C5	1.3586 (17)	C14—C15	1.370 (2)
N2—C10	1.3591 (18)	C14—H14	0.9500
N3—C15	1.3643 (17)	C15—H15	0.9500
N1'—C2	1.3732 (18)	C16—Cl3	1.7528 (15)
N2'—C7	1.3750 (18)	C16—Cl4	1.7603 (15)
N3'—C12	1.3700 (17)	C16—Cl2	1.7637 (16)
C2—C3	1.373 (2)	C16—D16	1.0000
C2—H2	0.9500	C17—Cl7	1.7596 (15)
C3—C4	1.393 (2)	C17—Cl6	1.7618 (15)
C3—H3	0.9500	C17—Cl5	1.7634 (16)
C4—C5	1.371 (2)	C17—D17	1.0000
C4—H4	0.9500		
			
O3—Si1—O2	175.08 (5)	N1—C5—H5	120.9
O3—Si1—O6	95.75 (5)	C4—C5—H5	120.9
O2—Si1—O6	88.80 (5)	N2'—C7—C8	117.79 (13)
O3—Si1—O1	88.58 (5)	C8—C7—H7	121.1
O2—Si1—O1	86.99 (5)	C7—C8—C9	120.70 (13)
O6—Si1—O1	174.48 (5)	C7—C8—H8	119.7
O3—Si1—O4	87.03 (4)	C9—C8—H8	119.7
O2—Si1—O4	91.19 (5)	C10—C9—C8	120.34 (13)
O6—Si1—O4	89.11 (4)	C10—C9—H9	119.8
O1—Si1—O4	94.54 (5)	C8—C9—H9	119.8
O3—Si1—O5	87.33 (5)	N2—C10—C9	117.44 (13)
O2—Si1—O5	94.77 (5)	N2—C10—H10	121.3
O6—Si1—O5	87.24 (4)	C9—C10—H10	121.3
O1—Si1—O5	89.56 (5)	N3'—C12—C13	117.96 (13)
O4—Si1—O5	172.94 (5)	C13—C12—H12	121.0
N1—O1—Si1	111.84 (8)	C12—C13—C14	120.20 (13)
N1'—O2—Si1	112.63 (8)	C12—C13—H13	119.9
N2—O3—Si1	111.55 (8)	C14—C13—H13	119.9
N2'—O4—Si1	111.98 (8)	C15—C14—C13	120.37 (13)
N3—O5—Si1	111.66 (8)	C15—C14—H14	119.8
N3'—O6—Si1	112.15 (8)	C13—C14—H14	119.8
O1—N1—C5	123.22 (11)	N3—C15—C14	118.12 (13)
O3—N2—C10	122.40 (11)	N3—C15—H15	120.9
O5—N3—C15	123.49 (12)	C14—C15—H15	120.9
O2—N1'—C2	125.51 (12)	Cl3—C16—Cl4	111.02 (9)
O4—N2'—C7	125.60 (12)	Cl3—C16—Cl2	110.35 (8)
O6—N3'—C12	124.24 (11)	Cl4—C16—Cl2	110.31 (8)
C3—C2—N1'	118.02 (13)	Cl3—C16—D16	108.4
C3—C2—H2	121.0	Cl4—C16—D16	108.4
C2—C3—C4	121.11 (13)	Cl2—C16—D16	108.4
C2—C3—H3	119.4	Cl7—C17—Cl6	110.37 (8)
C4—C3—H3	119.4	Cl7—C17—Cl5	110.40 (8)
C5—C4—C3	119.54 (13)	Cl6—C17—Cl5	110.72 (8)
C5—C4—H4	120.2	Cl7—C17—D17	108.4
C3—C4—H4	120.2	Cl6—C17—D17	108.4
N1—C5—C4	118.25 (13)	Cl5—C17—D17	108.4
			
O3—Si1—O1—N1	−177.57 (8)	O5—Si1—O6—N3'	1.60 (9)
O2—Si1—O1—N1	4.57 (8)	Si1—O1—N1—C5	177.54 (10)
O4—Si1—O1—N1	95.53 (8)	Si1—O3—N2—C10	173.83 (10)
O5—Si1—O1—N1	−90.23 (8)	Si1—O5—N3—C15	−179.99 (10)
O6—Si1—O2—N1'	171.18 (9)	Si1—O2—N1'—C2	−175.03 (11)
O1—Si1—O2—N1'	−5.25 (9)	Si1—O4—N2'—C7	−170.73 (11)
O4—Si1—O2—N1'	−99.73 (9)	Si1—O6—N3'—C12	178.86 (10)
O5—Si1—O2—N1'	84.05 (9)	O2—N1'—C2—C3	−178.96 (12)
O6—Si1—O3—N2	97.69 (8)	N1'—C2—C3—C4	−0.4 (2)
O1—Si1—O3—N2	−85.74 (8)	C2—C3—C4—C5	0.1 (2)
O4—Si1—O3—N2	8.88 (8)	O1—N1—C5—C4	−179.05 (12)
O5—Si1—O3—N2	−175.36 (8)	C3—C4—C5—N1	−0.7 (2)
O3—Si1—O4—N2'	−9.74 (8)	O4—N2'—C7—C8	179.67 (12)
O2—Si1—O4—N2'	165.67 (8)	N2'—C7—C8—C9	1.5 (2)
O6—Si1—O4—N2'	−105.55 (9)	C7—C8—C9—C10	−1.9 (2)
O1—Si1—O4—N2'	78.60 (9)	O3—N2—C10—C9	−178.23 (12)
O3—Si1—O5—N3	−96.91 (9)	C8—C9—C10—N2	0.2 (2)
O2—Si1—O5—N3	87.55 (9)	O6—N3'—C12—C13	179.45 (12)
O6—Si1—O5—N3	−1.02 (9)	N3'—C12—C13—C14	−0.6 (2)
O1—Si1—O5—N3	174.49 (8)	C12—C13—C14—C15	0.2 (2)
O3—Si1—O6—N3'	88.63 (9)	O5—N3—C15—C14	179.28 (12)
O2—Si1—O6—N3'	−93.24 (9)	C13—C14—C15—N3	0.5 (2)
O4—Si1—O6—N3'	175.55 (9)		

### (II) Tris[1-oxopyridine-2-olato(1-)]silicon(IV) chloride acetonitrile unknown solvate Crystal data

**Table:**

C_15_H_12_N_3_O_6_Si^+^·Cl^−^	*Z* = 2
*M**_r_* = 393.82	*F*(000) = 404
Triclinic, *P*1	*D*_x_ = 1.342 Mg m^−^^3^
*a* = 6.8347 (7) Å	Mo *K*α radiation, λ = 0.71073 Å
*b* = 11.1232 (12) Å	Cell parameters from 4067 reflections
*c* = 13.1513 (14) Å	θ = 2.5–36.4°
α = 90.479 (2)°	µ = 0.29 mm^−^^1^
β = 93.269 (2)°	*T* = 100 K
γ = 102.356 (2)°	Block, colorless
*V* = 974.85 (18) Å^3^	0.30 × 0.30 × 0.24 mm

### (II) Tris[1-oxopyridine-2-olato(1-)]silicon(IV) chloride acetonitrile unknown solvate Data collection

**Table:**

Bruker SMART APEXII CCD Platform diffractometer	6677 reflections with *I* > 2σ(*I*)
Radiation source: fine-focus sealed tube	*R*_int_ = 0.037
ω scans	θ_max_ = 38.5°, θ_min_ = 1.6°
Absorption correction: multi-scan (SADABS; Sheldrick, 2014)	*h* = −11→11
*T*_min_ = 0.645, *T*_max_ = 0.748	*k* = −19→19
27002 measured reflections	*l* = −22→22
10311 independent reflections	

### (II) Tris[1-oxopyridine-2-olato(1-)]silicon(IV) chloride acetonitrile unknown solvate Refinement

**Table:**

Refinement on *F*^2^	Primary atom site location: structure-invariant direct methods
Least-squares matrix: full	Secondary atom site location: difference Fourier map
*R*[*F*^2^ > 2σ(*F*^2^)] = 0.050	Hydrogen site location: inferred from neighbouring sites
*wR*(*F*^2^) = 0.132	H-atom parameters constrained
*S* = 1.03	*w* = 1/[σ^2^(*F*_o_^2^) + (0.0537*P*)^2^ + 0.1944*P*] where *P* = (*F*_o_^2^ + 2*F*_c_^2^)/3
10311 reflections	(Δ/σ)_max_ = 0.001
238 parameters	Δρ_max_ = 0.45 e Å^−^^3^
0 restraints	Δρ_min_ = −0.45 e Å^−^^3^

### (II) Tris[1-oxopyridine-2-olato(1-)]silicon(IV) chloride acetonitrile unknown solvate Special details

**Table:**

Geometry. All e.s.d.'s (except the e.s.d. in the dihedral angle between two l.s. planes) are estimated using the full covariance matrix. The cell e.s.d.'s are taken into account individually in the estimation of e.s.d.'s in distances, angles and torsion angles; correlations between e.s.d.'s in cell parameters are only used when they are defined by crystal symmetry. An approximate (isotropic) treatment of cell e.s.d.'s is used for estimating e.s.d.'s involving l.s. planes.
Refinement. Highly disordered solvent, located in two independent channels along [100], was unable to be modeled. Reflection contributions from this solvent were fixed and added to the calculated structure factors using the SQUEEZE function of program *PLATON* (Spek, 2009), which determined there to be 54 electrons in 225 Å^3^ accounted for per unit cell (25 electrons in 109 Å^3^ in one channel, and 29 electrons in 115 Å^3^ in the other). Because the exact identity and amount of solvent were unknown, no solvent was included in the atom list or molecular formula. Thus all calculated quantities that derive from the molecular formula (*e.g.*, *F*(000), density, molecular weight, *etc*.) are known to be incorrect.The pyridine portions of the oxopyridinone ligands are modeled as disordered with the planar flips of themselves (0.55:0.45, 0.60:0.40, and 0.61:0.39, for rings containing N1, N2, and N3, respectively). The disorders were modeled by refining the nitrogen/carbon ratios at the six particular atom sites, and refining the same ratio variable for pairs that were on the same ligand. Atoms at each of the six sites were constrained to be isopositional and to have equivalent anisotropic displacement parameters.

### (II) Tris[1-oxopyridine-2-olato(1-)]silicon(IV) chloride acetonitrile unknown solvate Fractional atomic coordinates and isotropic or equivalent isotropic displacement parameters (Å^2^)

**Table:**

	*x*	*y*	*z*	*U*_iso_*/*U*_eq_	Occ. (<1)
Si1	0.12051 (5)	0.29394 (3)	0.75582 (3)	0.01923 (7)	
Cl1	−0.28682 (5)	0.69679 (3)	0.72923 (3)	0.02991 (8)	
O1	0.24002 (13)	0.45107 (8)	0.77479 (7)	0.02124 (16)	
O2	0.07949 (13)	0.29060 (9)	0.88854 (7)	0.02376 (18)	
O3	0.35872 (12)	0.25406 (8)	0.77135 (7)	0.02194 (17)	
O4	0.01261 (14)	0.13330 (9)	0.74814 (8)	0.02702 (19)	
O5	0.15614 (13)	0.30843 (9)	0.62308 (7)	0.02244 (17)	
O6	−0.12215 (13)	0.32303 (9)	0.72917 (7)	0.02439 (18)	
N1	0.25249 (16)	0.48589 (11)	0.87312 (9)	0.0221 (2)	0.555 (13)
N2	0.15376 (18)	0.06613 (11)	0.75335 (10)	0.0268 (3)	0.604 (14)
N3	−0.00733 (16)	0.33084 (10)	0.57121 (8)	0.0206 (2)	0.611 (13)
C1	0.16641 (16)	0.39789 (12)	0.93573 (9)	0.0230 (2)	0.555 (13)
C6	0.34428 (18)	0.13298 (11)	0.76534 (10)	0.0247 (2)	0.604 (14)
C11	−0.15960 (16)	0.34008 (11)	0.63017 (9)	0.0221 (2)	0.611 (13)
C1'	0.25249 (16)	0.48589 (11)	0.87312 (9)	0.0221 (2)	0.445 (13)
C6'	0.15376 (18)	0.06613 (11)	0.75335 (10)	0.0268 (3)	0.396 (14)
C11'	−0.00733 (16)	0.33084 (10)	0.57121 (8)	0.0206 (2)	0.389 (13)
N1'	0.16641 (16)	0.39789 (12)	0.93573 (9)	0.0230 (2)	0.445 (13)
N2'	0.34428 (18)	0.13298 (11)	0.76534 (10)	0.0247 (2)	0.396 (14)
N3'	−0.15960 (16)	0.34008 (11)	0.63017 (9)	0.0221 (2)	0.389 (13)
C2	0.17350 (19)	0.41721 (15)	1.03917 (10)	0.0288 (3)	
H2	0.1159	0.3534	1.0829	0.035*	
C3	0.2666 (2)	0.53190 (17)	1.07748 (11)	0.0354 (3)	
H3	0.2733	0.5481	1.1488	0.042*	
C4	0.3511 (2)	0.62439 (15)	1.01246 (12)	0.0336 (3)	
H4	0.4119	0.7040	1.0394	0.040*	
C5	0.34709 (19)	0.60096 (13)	0.90880 (11)	0.0279 (3)	
H5	0.4078	0.6625	0.8639	0.034*	
C7	0.5050 (2)	0.07662 (16)	0.77229 (14)	0.0419 (4)	
H7	0.6388	0.1233	0.7807	0.050*	
C8	0.4648 (4)	−0.0500 (2)	0.7667 (2)	0.0644 (6)	
H8	0.5723	−0.0919	0.7713	0.077*	
C9	0.2670 (4)	−0.11734 (17)	0.75419 (19)	0.0637 (6)	
H9	0.2414	−0.2047	0.7505	0.076*	
C10	0.1110 (3)	−0.05921 (14)	0.74725 (15)	0.0438 (4)	
H10	−0.0236	−0.1046	0.7384	0.053*	
C12	−0.33579 (19)	0.36479 (14)	0.58824 (12)	0.0304 (3)	
H12	−0.4423	0.3720	0.6297	0.037*	
C13	−0.3527 (2)	0.37871 (14)	0.48454 (12)	0.0357 (3)	
H13	−0.4730	0.3948	0.4534	0.043*	
C14	−0.1941 (2)	0.36936 (13)	0.42463 (11)	0.0327 (3)	
H14	−0.2068	0.3803	0.3532	0.039*	
C15	−0.0198 (2)	0.34451 (12)	0.46814 (10)	0.0264 (2)	
H15	0.0882	0.3371	0.4279	0.032*	

### (II) Tris[1-oxopyridine-2-olato(1-)]silicon(IV) chloride acetonitrile unknown solvate Atomic displacement parameters (Å^2^)

**Table:**

	*U*^11^	*U*^22^	*U*^33^	*U*^12^	*U*^13^	*U*^23^
Si1	0.01513 (13)	0.02434 (16)	0.01861 (15)	0.00486 (11)	0.00187 (11)	0.00053 (11)
Cl1	0.02417 (14)	0.03503 (17)	0.03014 (17)	0.00432 (12)	0.00548 (11)	0.00471 (13)
O1	0.0231 (4)	0.0246 (4)	0.0161 (4)	0.0050 (3)	0.0017 (3)	−0.0003 (3)
O2	0.0223 (4)	0.0297 (4)	0.0203 (4)	0.0069 (3)	0.0041 (3)	0.0028 (3)
O3	0.0170 (4)	0.0237 (4)	0.0257 (4)	0.0060 (3)	0.0001 (3)	−0.0006 (3)
O4	0.0209 (4)	0.0258 (4)	0.0335 (5)	0.0039 (3)	−0.0018 (4)	0.0007 (4)
O5	0.0179 (4)	0.0313 (4)	0.0189 (4)	0.0073 (3)	0.0006 (3)	−0.0013 (3)
O6	0.0177 (4)	0.0354 (5)	0.0218 (4)	0.0091 (3)	0.0021 (3)	0.0042 (4)
N1	0.0167 (4)	0.0310 (6)	0.0206 (5)	0.0102 (4)	−0.0003 (4)	−0.0041 (4)
N2	0.0251 (5)	0.0252 (5)	0.0293 (6)	0.0052 (4)	−0.0039 (4)	−0.0045 (4)
N3	0.0188 (4)	0.0231 (5)	0.0196 (5)	0.0041 (4)	−0.0009 (4)	−0.0017 (4)
C1	0.0170 (4)	0.0368 (6)	0.0182 (5)	0.0129 (4)	0.0007 (4)	−0.0009 (4)
C6	0.0230 (5)	0.0255 (5)	0.0265 (6)	0.0081 (4)	−0.0007 (4)	−0.0029 (4)
C11	0.0179 (4)	0.0277 (5)	0.0211 (5)	0.0061 (4)	0.0005 (4)	0.0013 (4)
C1'	0.0167 (4)	0.0310 (6)	0.0206 (5)	0.0102 (4)	−0.0003 (4)	−0.0041 (4)
C6'	0.0251 (5)	0.0252 (5)	0.0293 (6)	0.0052 (4)	−0.0039 (4)	−0.0045 (4)
C11'	0.0188 (4)	0.0231 (5)	0.0196 (5)	0.0041 (4)	−0.0009 (4)	−0.0017 (4)
N1'	0.0170 (4)	0.0368 (6)	0.0182 (5)	0.0129 (4)	0.0007 (4)	−0.0009 (4)
N2'	0.0230 (5)	0.0255 (5)	0.0265 (6)	0.0081 (4)	−0.0007 (4)	−0.0029 (4)
N3'	0.0179 (4)	0.0277 (5)	0.0211 (5)	0.0061 (4)	0.0005 (4)	0.0013 (4)
C2	0.0193 (5)	0.0548 (9)	0.0174 (5)	0.0194 (5)	0.0010 (4)	−0.0006 (5)
C3	0.0226 (6)	0.0654 (10)	0.0240 (6)	0.0243 (6)	−0.0038 (5)	−0.0141 (6)
C4	0.0215 (6)	0.0463 (8)	0.0354 (8)	0.0155 (6)	−0.0073 (5)	−0.0177 (6)
C5	0.0185 (5)	0.0335 (7)	0.0335 (7)	0.0104 (5)	−0.0019 (5)	−0.0067 (5)
C7	0.0306 (7)	0.0450 (9)	0.0548 (11)	0.0208 (7)	−0.0046 (7)	−0.0084 (8)
C8	0.0654 (13)	0.0514 (11)	0.0862 (17)	0.0405 (11)	−0.0166 (12)	−0.0175 (11)
C9	0.0809 (16)	0.0280 (8)	0.0836 (17)	0.0226 (9)	−0.0216 (13)	−0.0183 (9)
C10	0.0499 (10)	0.0241 (7)	0.0529 (11)	0.0030 (6)	−0.0146 (8)	−0.0060 (7)
C12	0.0190 (5)	0.0378 (7)	0.0359 (7)	0.0098 (5)	−0.0014 (5)	0.0046 (6)
C13	0.0337 (7)	0.0370 (7)	0.0363 (8)	0.0117 (6)	−0.0141 (6)	0.0001 (6)
C14	0.0476 (8)	0.0264 (6)	0.0225 (6)	0.0076 (6)	−0.0100 (6)	−0.0020 (5)
C15	0.0352 (7)	0.0236 (6)	0.0199 (6)	0.0051 (5)	0.0020 (5)	−0.0032 (4)

### (II) Tris[1-oxopyridine-2-olato(1-)]silicon(IV) chloride acetonitrile unknown solvate Geometric parameters (Å, º)

**Table:**

Si1—O1	1.7727 (10)	C6'—N2'	1.3542 (17)
Si1—O6	1.7729 (9)	C6'—C10	1.3626 (19)
Si1—O3	1.7782 (9)	C11'—N3'	1.3533 (15)
Si1—O5	1.7803 (10)	C11'—C15	1.3651 (17)
Si1—O4	1.7808 (10)	N1'—C2	1.3719 (17)
Si1—O2	1.7830 (10)	N2'—C7	1.3755 (18)
O1—C1'	1.3396 (14)	N3'—C12	1.3777 (16)
O1—N1	1.3396 (14)	C2—C3	1.375 (2)
O2—N1'	1.3433 (16)	C2—H2	0.9500
O2—C1	1.3433 (16)	C3—C4	1.393 (2)
O3—N2'	1.3305 (15)	C3—H3	0.9500
O3—C6	1.3305 (15)	C4—C5	1.383 (2)
O4—C6'	1.3399 (15)	C4—H4	0.9500
O4—N2	1.3399 (15)	C5—H5	0.9500
O5—C11'	1.3457 (13)	C7—C8	1.376 (3)
O5—N3	1.3457 (13)	C7—H7	0.9500
O6—N3'	1.3356 (14)	C8—C9	1.398 (3)
O6—C11	1.3356 (14)	C8—H8	0.9500
N1—C1	1.3434 (17)	C9—C10	1.360 (3)
N1—C5	1.3703 (18)	C9—H9	0.9500
N2—C6	1.3542 (17)	C10—H10	0.9500
N2—C10	1.3626 (19)	C12—C13	1.375 (2)
N3—C11	1.3533 (15)	C12—H12	0.9500
N3—C15	1.3651 (17)	C13—C14	1.397 (2)
C1—C2	1.3719 (17)	C13—H13	0.9500
C6—C7	1.3755 (18)	C14—C15	1.374 (2)
C11—C12	1.3777 (16)	C14—H14	0.9500
C1'—N1'	1.3434 (17)	C15—H15	0.9500
C1'—C5	1.3703 (18)		
			
O1—Si1—O6	94.90 (5)	N3'—C11'—C15	122.31 (11)
O1—Si1—O3	89.28 (4)	O2—N1'—C1'	114.28 (10)
O6—Si1—O3	174.02 (5)	O2—N1'—C2	123.88 (12)
O1—Si1—O5	89.48 (4)	C1'—N1'—C2	121.81 (13)
O6—Si1—O5	87.36 (4)	O3—N2'—C6'	114.34 (10)
O3—Si1—O5	88.40 (4)	O3—N2'—C7	124.56 (13)
O1—Si1—O4	174.42 (5)	C6'—N2'—C7	121.09 (13)
O6—Si1—O4	88.77 (5)	O6—N3'—C11'	114.24 (10)
O3—Si1—O4	87.38 (4)	O6—N3'—C12	124.74 (11)
O5—Si1—O4	94.90 (5)	C11'—N3'—C12	121.02 (12)
O1—Si1—O2	87.28 (4)	N1'—C2—C3	117.86 (14)
O6—Si1—O2	89.96 (4)	C1—C2—C3	117.86 (14)
O3—Si1—O2	94.51 (4)	C1—C2—H2	121.1
O5—Si1—O2	175.60 (5)	C3—C2—H2	121.1
O4—Si1—O2	88.53 (5)	C2—C3—C4	120.44 (13)
C1'—O1—Si1	111.97 (8)	C2—C3—H3	119.8
N1—O1—Si1	111.97 (8)	C4—C3—H3	119.8
N1'—O2—Si1	111.62 (8)	C5—C4—C3	120.34 (14)
C1—O2—Si1	111.62 (8)	C5—C4—H4	119.8
N2'—O3—Si1	112.12 (8)	C3—C4—H4	119.8
C6—O3—Si1	112.12 (8)	C1'—C5—C4	117.69 (14)
C6'—O4—Si1	111.55 (8)	N1—C5—C4	117.69 (14)
N2—O4—Si1	111.55 (8)	N1—C5—H5	121.2
C11'—O5—Si1	111.74 (7)	C4—C5—H5	121.2
N3—O5—Si1	111.74 (7)	N2'—C7—C8	117.54 (17)
N3'—O6—Si1	112.42 (7)	C6—C7—C8	117.54 (17)
C11—O6—Si1	112.42 (7)	C6—C7—H7	121.2
O1—N1—C1	114.61 (11)	C8—C7—H7	121.2
O1—N1—C5	123.59 (12)	C7—C8—C9	120.50 (17)
C1—N1—C5	121.79 (12)	C7—C8—H8	119.7
O4—N2—C6	114.51 (11)	C9—C8—H8	119.7
O4—N2—C10	123.25 (13)	C10—C9—C8	120.72 (17)
C6—N2—C10	122.24 (13)	C10—C9—H9	119.6
O5—N3—C11	114.23 (10)	C8—C9—H9	119.6
O5—N3—C15	123.46 (11)	C9—C10—N2	117.90 (17)
C11—N3—C15	122.31 (11)	C9—C10—C6'	117.90 (17)
O2—C1—N1	114.28 (10)	C9—C10—H10	121.1
O2—C1—C2	123.88 (12)	N2—C10—H10	121.1
N1—C1—C2	121.81 (13)	C13—C12—C11	117.91 (13)
O3—C6—N2	114.34 (10)	C13—C12—N3'	117.91 (13)
O3—C6—C7	124.56 (13)	C13—C12—H12	121.0
N2—C6—C7	121.09 (13)	C11—C12—H12	121.0
O6—C11—N3	114.24 (10)	C12—C13—C14	120.48 (13)
O6—C11—C12	124.74 (11)	C12—C13—H13	119.8
N3—C11—C12	121.02 (12)	C14—C13—H13	119.8
O1—C1'—N1'	114.61 (11)	C15—C14—C13	120.58 (13)
O1—C1'—C5	123.59 (12)	C15—C14—H14	119.7
N1'—C1'—C5	121.79 (12)	C13—C14—H14	119.7
O4—C6'—N2'	114.51 (11)	C11'—C15—C14	117.70 (13)
O4—C6'—C10	123.25 (13)	N3—C15—C14	117.70 (13)
N2'—C6'—C10	122.24 (13)	N3—C15—H15	121.1
O5—C11'—N3'	114.23 (10)	C14—C15—H15	121.1
O5—C11'—C15	123.46 (11)		
			
O6—Si1—O1—C1'	93.66 (8)	O5—N3—C11—O6	−1.19 (15)
O3—Si1—O1—C1'	−90.62 (8)	C15—N3—C11—O6	179.43 (11)
O5—Si1—O1—C1'	−179.03 (7)	O5—N3—C11—C12	178.96 (12)
O2—Si1—O1—C1'	3.93 (7)	C15—N3—C11—C12	−0.4 (2)
O6—Si1—O1—N1	93.66 (8)	Si1—O1—C1'—N1'	−2.53 (12)
O3—Si1—O1—N1	−90.62 (8)	Si1—O1—C1'—C5	176.61 (9)
O5—Si1—O1—N1	−179.03 (7)	Si1—O4—C6'—N2'	1.35 (15)
O2—Si1—O1—N1	3.93 (7)	Si1—O4—C6'—C10	−178.70 (13)
O1—Si1—O2—N1'	−4.51 (7)	Si1—O5—C11'—N3'	1.34 (13)
O6—Si1—O2—N1'	−99.42 (8)	Si1—O5—C11'—C15	−179.29 (10)
O3—Si1—O2—N1'	84.55 (8)	Si1—O2—N1'—C1'	4.17 (12)
O4—Si1—O2—N1'	171.81 (8)	Si1—O2—N1'—C2	−174.11 (9)
O1—Si1—O2—C1	−4.51 (7)	O1—C1'—N1'—O2	−1.10 (14)
O6—Si1—O2—C1	−99.42 (8)	C5—C1'—N1'—O2	179.75 (10)
O3—Si1—O2—C1	84.55 (8)	O1—C1'—N1'—C2	177.22 (10)
O4—Si1—O2—C1	171.81 (8)	C5—C1'—N1'—C2	−1.93 (17)
O1—Si1—O3—N2'	178.35 (8)	Si1—O3—N2'—C6'	−2.72 (14)
O5—Si1—O3—N2'	−92.16 (9)	Si1—O3—N2'—C7	178.47 (13)
O4—Si1—O3—N2'	2.82 (9)	O4—C6'—N2'—O3	0.90 (17)
O2—Si1—O3—N2'	91.13 (9)	C10—C6'—N2'—O3	−179.05 (14)
O1—Si1—O3—C6	178.35 (8)	O4—C6'—N2'—C7	179.75 (14)
O5—Si1—O3—C6	−92.16 (9)	C10—C6'—N2'—C7	−0.2 (2)
O4—Si1—O3—C6	2.82 (9)	Si1—O6—N3'—C11'	0.47 (13)
O2—Si1—O3—C6	91.13 (9)	Si1—O6—N3'—C12	−179.69 (11)
O6—Si1—O4—C6'	173.11 (9)	O5—C11'—N3'—O6	−1.19 (15)
O3—Si1—O4—C6'	−2.31 (9)	C15—C11'—N3'—O6	179.43 (11)
O5—Si1—O4—C6'	85.86 (9)	O5—C11'—N3'—C12	178.96 (12)
O2—Si1—O4—C6'	−96.90 (9)	C15—C11'—N3'—C12	−0.4 (2)
O6—Si1—O4—N2	173.11 (9)	O2—N1'—C2—C3	−179.73 (10)
O3—Si1—O4—N2	−2.31 (9)	C1'—N1'—C2—C3	2.12 (17)
O5—Si1—O4—N2	85.86 (9)	O2—C1—C2—C3	−179.73 (10)
O2—Si1—O4—N2	−96.90 (9)	N1—C1—C2—C3	2.12 (17)
O1—Si1—O5—C11'	−95.80 (8)	N1'—C2—C3—C4	−0.29 (18)
O6—Si1—O5—C11'	−0.87 (8)	C1—C2—C3—C4	−0.29 (18)
O3—Si1—O5—C11'	174.91 (8)	C2—C3—C4—C5	−1.73 (19)
O4—Si1—O5—C11'	87.67 (8)	O1—C1'—C5—C4	−179.23 (10)
O1—Si1—O5—N3	−95.80 (8)	N1'—C1'—C5—C4	−0.15 (17)
O6—Si1—O5—N3	−0.87 (8)	O1—N1—C5—C4	−179.23 (10)
O3—Si1—O5—N3	174.91 (8)	C1—N1—C5—C4	−0.15 (17)
O4—Si1—O5—N3	87.67 (8)	C3—C4—C5—C1'	1.94 (18)
O1—Si1—O6—N3'	89.47 (9)	C3—C4—C5—N1	1.94 (18)
O5—Si1—O6—N3'	0.22 (9)	O3—N2'—C7—C8	178.70 (17)
O4—Si1—O6—N3'	−94.73 (9)	C6'—N2'—C7—C8	0.0 (3)
O2—Si1—O6—N3'	176.74 (9)	O3—C6—C7—C8	178.70 (17)
O1—Si1—O6—C11	89.47 (9)	N2—C6—C7—C8	0.0 (3)
O5—Si1—O6—C11	0.22 (9)	N2'—C7—C8—C9	0.1 (3)
O4—Si1—O6—C11	−94.73 (9)	C6—C7—C8—C9	0.1 (3)
O2—Si1—O6—C11	176.74 (9)	C7—C8—C9—C10	0.1 (4)
Si1—O1—N1—C1	−2.53 (12)	C8—C9—C10—N2	−0.3 (3)
Si1—O1—N1—C5	176.61 (9)	C8—C9—C10—C6'	−0.3 (3)
Si1—O4—N2—C6	1.35 (15)	O4—N2—C10—C9	−179.58 (17)
Si1—O4—N2—C10	−178.70 (13)	C6—N2—C10—C9	0.4 (3)
Si1—O5—N3—C11	1.34 (13)	O4—C6'—C10—C9	−179.58 (17)
Si1—O5—N3—C15	−179.29 (10)	N2'—C6'—C10—C9	0.4 (3)
Si1—O2—C1—N1	4.17 (12)	O6—C11—C12—C13	−179.28 (13)
Si1—O2—C1—C2	−174.11 (9)	N3—C11—C12—C13	0.6 (2)
O1—N1—C1—O2	−1.10 (14)	O6—N3'—C12—C13	−179.28 (13)
C5—N1—C1—O2	179.75 (10)	C11'—N3'—C12—C13	0.6 (2)
O1—N1—C1—C2	177.22 (10)	C11—C12—C13—C14	−0.8 (2)
C5—N1—C1—C2	−1.93 (17)	N3'—C12—C13—C14	−0.8 (2)
Si1—O3—C6—N2	−2.72 (14)	C12—C13—C14—C15	0.9 (2)
Si1—O3—C6—C7	178.47 (13)	O5—C11'—C15—C14	−178.84 (12)
O4—N2—C6—O3	0.90 (17)	N3'—C11'—C15—C14	0.48 (19)
C10—N2—C6—O3	−179.05 (14)	O5—N3—C15—C14	−178.84 (12)
O4—N2—C6—C7	179.75 (14)	C11—N3—C15—C14	0.48 (19)
C10—N2—C6—C7	−0.2 (2)	C13—C14—C15—C11'	−0.7 (2)
Si1—O6—C11—N3	0.47 (13)	C13—C14—C15—N3	−0.7 (2)
Si1—O6—C11—C12	−179.69 (11)		

### (III) fac-Tris[1-oxopyridine-2-thiolato(1-)]silicon(IV) chloride chloroform-d1 disolvate Crystal data

**Table:**

C_15_H_12_N_3_O_3_S_3_Si^+^·Cl^−^·2CDCl_3_	Mo *K*α radiation, λ = 0.71073 Å
*M**_r_* = 682.74	Cell parameters from 4002 reflections
Cubic, *P*2_1_3	θ = 2.5–33.8°
*a* = 13.9483 (12) Å	µ = 1.03 mm^−^^1^
*V* = 2713.7 (7) Å^3^	*T* = 100 K
*Z* = 4	Tetrahedron, pale yellow
*F*(000) = 1368	0.18 × 0.18 × 0.18 mm
*D*_x_ = 1.671 Mg m^−^^3^	

### (III) fac-Tris[1-oxopyridine-2-thiolato(1-)]silicon(IV) chloride chloroform-d1 disolvate Data collection

**Table:**

Bruker SMART APEXII CCD Platform diffractometer	4360 reflections with *I* > 2σ(*I*)
Radiation source: fine-focus sealed tube	*R*_int_ = 0.059
ω scans	θ_max_ = 38.5°, θ_min_ = 2.1°
Absorption correction: multi-scan (SADABS; Sheldrick, 2014)	*h* = −24→24
*T*_min_ = 0.681, *T*_max_ = 0.748	*k* = −23→24
66318 measured reflections	*l* = −23→24
5067 independent reflections	

### (III) fac-Tris[1-oxopyridine-2-thiolato(1-)]silicon(IV) chloride chloroform-d1 disolvate Refinement

**Table:**

Refinement on *F*^2^	Secondary atom site location: difference Fourier map
Least-squares matrix: full	Hydrogen site location: inferred from neighbouring sites
*R*[*F*^2^ > 2σ(*F*^2^)] = 0.037	H-atom parameters constrained
*wR*(*F*^2^) = 0.088	*w* = 1/[σ^2^(*F*_o_^2^) + (0.0387*P*)^2^ + 1.2622*P*] where *P* = (*F*_o_^2^ + 2*F*_c_^2^)/3
*S* = 1.03	(Δ/σ)_max_ = 0.001
5067 reflections	Δρ_max_ = 0.88 e Å^−^^3^
103 parameters	Δρ_min_ = −0.67 e Å^−^^3^
0 restraints	Absolute structure: Flack *x* determined using 1775 quotients [(*I*^+^)-(*I*^-^)]/[(*I*^+^)+(*I*^-^)] (Parsons *et al.*, 2013)
Primary atom site location: structure-invariant direct methods	Absolute structure parameter: −0.018 (18)

### (III) fac-Tris[1-oxopyridine-2-thiolato(1-)]silicon(IV) chloride chloroform-d1 disolvate Special details

**Table:**

Geometry. All e.s.d.'s (except the e.s.d. in the dihedral angle between two l.s. planes) are estimated using the full covariance matrix. The cell e.s.d.'s are taken into account individually in the estimation of e.s.d.'s in distances, angles and torsion angles; correlations between e.s.d.'s in cell parameters are only used when they are defined by crystal symmetry. An approximate (isotropic) treatment of cell e.s.d.'s is used for estimating e.s.d.'s involving l.s. planes.

### (III) fac-Tris[1-oxopyridine-2-thiolato(1-)]silicon(IV) chloride chloroform-d1 disolvate Fractional atomic coordinates and isotropic or equivalent isotropic displacement parameters (Å^2^)

**Table:**

	*x*	*y*	*z*	*U*_iso_*/*U*_eq_	
Cl1	0.47976 (3)	−0.02024 (3)	0.52024 (3)	0.01864 (13)	
Si1	0.48307 (3)	0.48307 (3)	0.48307 (3)	0.01248 (14)	
S1	0.47110 (3)	0.49896 (3)	0.64425 (3)	0.01526 (8)	
O1	0.45832 (11)	0.35879 (9)	0.49722 (10)	0.0153 (2)	
N1	0.46124 (12)	0.31975 (11)	0.58645 (11)	0.0138 (2)	
C1	0.46796 (14)	0.37754 (13)	0.66485 (13)	0.0146 (3)	
C2	0.47203 (16)	0.33345 (14)	0.75505 (14)	0.0190 (3)	
H2	0.4772	0.3713	0.8114	0.023*	
C3	0.46853 (18)	0.23462 (15)	0.76169 (15)	0.0220 (4)	
H3	0.4708	0.2045	0.8228	0.026*	
C4	0.46163 (15)	0.17883 (14)	0.67861 (15)	0.0187 (3)	
H4	0.4592	0.1109	0.6831	0.022*	
C5	0.45834 (14)	0.22276 (13)	0.59068 (14)	0.0161 (3)	
H5	0.4541	0.1858	0.5337	0.019*	
C6	0.30918 (15)	0.30918 (15)	0.30918 (15)	0.0212 (6)	
D6	0.3506	0.3506	0.3506	0.025*	
Cl2	0.30061 (7)	0.19603 (5)	0.36357 (6)	0.04462 (19)	
C7	0.12879 (19)	0.37121 (19)	0.62879 (19)	0.0264 (7)	
D7	0.0874	0.4126	0.5874	0.032*	
Cl3	0.21080 (7)	0.44529 (6)	0.68782 (8)	0.0511 (2)	

### (III) fac-Tris[1-oxopyridine-2-thiolato(1-)]silicon(IV) chloride chloroform-d1 disolvate Atomic displacement parameters (Å^2^)

**Table:**

	*U*^11^	*U*^22^	*U*^33^	*U*^12^	*U*^13^	*U*^23^
Cl1	0.01864 (13)	0.01864 (13)	0.01864 (13)	0.00093 (14)	−0.00093 (14)	−0.00093 (14)
Si1	0.01248 (14)	0.01248 (14)	0.01248 (14)	−0.00028 (14)	−0.00028 (14)	−0.00028 (14)
S1	0.01940 (18)	0.01277 (16)	0.01360 (16)	−0.00101 (14)	0.00168 (14)	−0.00190 (13)
O1	0.0215 (6)	0.0125 (5)	0.0121 (5)	−0.0009 (4)	−0.0009 (4)	0.0005 (4)
N1	0.0148 (6)	0.0134 (6)	0.0132 (6)	0.0002 (5)	0.0003 (5)	0.0004 (5)
C1	0.0158 (7)	0.0144 (6)	0.0137 (6)	0.0004 (5)	0.0008 (5)	−0.0009 (5)
C2	0.0252 (9)	0.0184 (7)	0.0134 (7)	0.0009 (7)	0.0013 (7)	0.0004 (6)
C3	0.0300 (10)	0.0182 (8)	0.0176 (8)	0.0021 (8)	−0.0003 (7)	0.0049 (6)
C4	0.0218 (8)	0.0144 (7)	0.0201 (8)	0.0008 (6)	0.0004 (7)	0.0028 (6)
C5	0.0177 (7)	0.0130 (7)	0.0178 (7)	0.0005 (5)	−0.0005 (6)	−0.0001 (5)
C6	0.0212 (6)	0.0212 (6)	0.0212 (6)	−0.0019 (7)	−0.0019 (7)	−0.0019 (7)
Cl2	0.0654 (5)	0.0202 (3)	0.0482 (4)	0.0016 (3)	0.0125 (4)	0.0041 (3)
C7	0.0264 (7)	0.0264 (7)	0.0264 (7)	−0.0015 (8)	0.0015 (8)	−0.0015 (8)
Cl3	0.0493 (5)	0.0432 (4)	0.0610 (5)	−0.0147 (3)	−0.0130 (4)	−0.0089 (4)

### (III) fac-Tris[1-oxopyridine-2-thiolato(1-)]silicon(IV) chloride chloroform-d1 disolvate Geometric parameters (Å, º)

**Table:**

Si1—O1^i^	1.7784 (14)	C3—C4	1.399 (3)
Si1—O1^ii^	1.7784 (14)	C3—H3	0.9500
Si1—O1	1.7784 (14)	C4—C5	1.372 (3)
Si1—S1	2.2654 (7)	C4—H4	0.9500
Si1—S1^i^	2.2654 (7)	C5—H5	0.9500
Si1—S1^ii^	2.2654 (7)	C6—Cl2^i^	1.7552 (13)
S1—C1	1.7184 (19)	C6—Cl2^ii^	1.7552 (13)
O1—N1	1.359 (2)	C6—Cl2	1.7552 (13)
N1—C5	1.355 (2)	C6—D6	1.0000
N1—C1	1.362 (2)	C7—Cl3^iii^	1.7476 (16)
C1—C2	1.402 (3)	C7—Cl3^iv^	1.7476 (16)
C2—C3	1.383 (3)	C7—Cl3	1.7476 (16)
C2—H2	0.9500	C7—D7	1.0000
			
O1^i^—Si1—O1^ii^	86.60 (7)	C3—C2—H2	120.1
O1^i^—Si1—O1	86.59 (7)	C1—C2—H2	120.1
O1^ii^—Si1—O1	86.59 (7)	C2—C3—C4	120.09 (18)
O1^i^—Si1—S1	96.31 (5)	C2—C3—H3	120.0
O1^ii^—Si1—S1	174.00 (5)	C4—C3—H3	120.0
O1—Si1—S1	88.33 (4)	C5—C4—C3	119.64 (17)
O1^i^—Si1—S1^i^	88.33 (4)	C5—C4—H4	120.2
O1^ii^—Si1—S1^i^	96.31 (5)	C3—C4—H4	120.2
O1—Si1—S1^i^	174.00 (5)	N1—C5—C4	118.94 (17)
S1—Si1—S1^i^	89.04 (3)	N1—C5—H5	120.5
O1^i^—Si1—S1^ii^	174.00 (5)	C4—C5—H5	120.5
O1^ii^—Si1—S1^ii^	88.33 (4)	Cl2^i^—C6—Cl2^ii^	110.91 (11)
O1—Si1—S1^ii^	96.31 (5)	Cl2^i^—C6—Cl2	110.91 (11)
S1—Si1—S1^ii^	89.04 (3)	Cl2^ii^—C6—Cl2	110.91 (11)
S1^i^—Si1—S1^ii^	89.04 (3)	Cl2^i^—C6—D6	108.0
C1—S1—Si1	94.09 (6)	Cl2^ii^—C6—D6	108.0
N1—O1—Si1	119.10 (11)	Cl2—C6—D6	108.0
C5—N1—O1	116.05 (15)	Cl3^iii^—C7—Cl3^iv^	110.93 (14)
C5—N1—C1	123.93 (16)	Cl3^iii^—C7—Cl3	110.93 (14)
O1—N1—C1	120.02 (14)	Cl3^iv^—C7—Cl3	110.93 (14)
N1—C1—C2	117.64 (16)	Cl3^iii^—C7—D7	108.0
N1—C1—S1	116.80 (13)	Cl3^iv^—C7—D7	108.0
C2—C1—S1	125.56 (14)	Cl3—C7—D7	108.0
C3—C2—C1	119.75 (18)		
			
O1^i^—Si1—O1—N1	−108.75 (16)	Si1—S1—C1—N1	−7.82 (15)
O1^ii^—Si1—O1—N1	164.47 (14)	Si1—S1—C1—C2	171.97 (18)
S1—Si1—O1—N1	−12.32 (12)	N1—C1—C2—C3	−0.4 (3)
S1^ii^—Si1—O1—N1	76.53 (13)	S1—C1—C2—C3	179.79 (18)
Si1—O1—N1—C5	−168.92 (13)	C1—C2—C3—C4	0.5 (4)
Si1—O1—N1—C1	10.3 (2)	C2—C3—C4—C5	0.0 (4)
C5—N1—C1—C2	0.0 (3)	O1—N1—C5—C4	179.61 (17)
O1—N1—C1—C2	−179.14 (18)	C1—N1—C5—C4	0.5 (3)
C5—N1—C1—S1	179.76 (15)	C3—C4—C5—N1	−0.4 (3)
O1—N1—C1—S1	0.7 (2)		

Symmetry codes: (i) *y*, *z*, *x*; (ii) *z*, *x*, *y*; (iii) *z*−1/2, −*x*+1/2, −*y*+1; (iv) −*y*+1/2, −*z*+1, *x*+1/2.
